# Molecular characterization of the prevalent soil-transmitted helminths in Narathiwat Province, southern Thailand

**DOI:** 10.1371/journal.pone.0347339

**Published:** 2026-04-16

**Authors:** Saiwasan Buathong, Nantana Suwandittakul, Musleeha Chesor, Piyaorn Chornchoem, Mathirut Mungthin

**Affiliations:** 1 Department of Clinical Pathology, Faculty of Medicine Vajira Hospital, Navamindradhiraj University, Dusit, Bangkok, Thailand; 2 Department of Public Health and Health Promotion, College of Allied Health Sciences, Suan Sunandha Rajabhat University, Samut Songkhram, Thailand; 3 Faculty of Medicine, Princess of Naradhiwas University, Narathiwat, Thailand; 4 Department of Parasitology, Phramongkutklao College of Medicine, Bangkok, Thailand; Institute of Tropical Medicine: Instituut voor Tropische Geneeskunde, BELGIUM

## Abstract

Soil-transmitted helminths (STHs) are prevalent in tropical regions, including southern Thailand. However, limited data on their abundance in this area and molecular characteristics exist. This study evaluated 210 fecal specimens from the Narathiwat Province to determine the molecular profiles of STHs. Microscopic examination revealed that the predominant STHs were *Trichuris* spp. and *Ascaris* spp., with prevalences of 14.76% and 6.67%, respectively. Based on ITS1 sequencing, two haplotypes of *Ascaris* spp. were identified, classified as *A*. *lumbricoides* and *A*. *suum*. ITS1-based phylogeny revealed two distinct lineages: *A*. *lumbricoides*, which clustered with isolates from humans and primates, and *A*. *suum*, with genetic similarity to isolates from humans and animals. Classification based on the *A. lumbricoides cox1* gene revealed five distinct haplotypes, including haplotype G, which exhibited high genetic similarity to *A. suum* isolates from pigs. In *Trichuris* spp., 18S rRNA sequencing identified four haplotypes that clustered with the reference isolates from humans and formed lineages distinct from the *Trichuris* spp. that infect animals. Analysis of the *cox1* sequences of *T. trichiura* revealed four haplotypes clustering into distinct clades, which were genetically similar to reported human isolates but clearly differentiated from animal isolates. Pairwise F_ST_ analysis revealed remarkable genetic variations within the populations of *Ascaris* and *Trichuris* spp., indicating potential species complexity. These findings provide valuable insights into the prevalence, genetic diversity, and molecular epidemiology of STHs in southern Thailand, which are vital for guiding further research to inform the control and prevention approaches in this endemic area.

## Introduction

Soil-transmitted helminths (STHs) are parasitic worms that infect humans and animals and are typically transmitted through soil contaminated with infective eggs or larvae [1]. Such infections are common in regions with poor sanitation facilities, especially in tropical and subtropical areas such as Sub-Saharan, Southeast Asia, and South America. Major STHs include *Ascaris lumbricoides*, *Trichuris trichiura*, hookworm (*Ancylostoma duodenale* and *Necator americanus*), and *Strongyloides stercoralis* [1–3]. Transmission of STHs occurs through the ingestion of eggs or skin penetration by larvae. Although initial symptoms may be mild, STHs can cause serious health issues, including anemia, stunted growth, cognitive delays, and intestinal obstruction [4,5]. These outcomes arise because adult worms compete for host nutrients, with hookworms in particular causing significant blood loss [5]. Beyond their direct health impacts, STHs exhibit substantial zoonotic potential, as several species traditionally regarded as animal parasites are capable of infecting humans under conditions of close human–animal contact. For example, *T. suis* (pig whipworm) and *T. vulpis* (dog whipworm) have been reported infecting pig farmers and dog owners [6–9], and *T. trichiura*, commonly considered a human parasite, has also been detected in domestic animals [6,8,10]. However, recent molecular studies have increasingly highlighted substantial genetic diversity among *Trichuris* isolates infecting humans. Notably, a novel human-infective species, *T. incognita*, has recently been reported from human fecal samples in several African countries, including Côte d’Ivoire, Tanzania, and Uganda. These findings suggest that the taxonomy and host range of human-infecting *Trichuris* species may be more complex than previously recognized. [11,12]. Similarly, zoonotic hookworms such as *A. ceylanicum* infect both humans and dogs, while *N. americanus* and *A. duodenale* remain important human pathogens [13–16]. *A. lumbricoides* (human roundworm) and *A. suum* (pig roundworm) can infect both humans and pigs, particularly in settings with poor sanitation and frequent human–pig contact [17–20].

In addition, both *Ascaris* species have been detected in non-human primates such as chimpanzees and gibbons, further underscoring their broad host range [21,22]. Moreover, several studies suggest that the zoonotic potential of STHs may be more extensive than previously recognized. Genetic studies have demonstrated overlap between human- and animal-derived *Ascaris* isolates across Asia, Africa, and Latin America [23,24]. A recent systematic review reported evidence of cross-host transmission of *A. lumbricoides* and *T. trichiura* in sympatric environments, as confirmed by molecular analyses [24]. Regionally, *Ascaris* isolates from Thailand cluster within mitochondrial clades shared between pigs and humans, suggesting zoonotic transmission or shared evolutionary lineages [25]. Similarly, *T. trichiura* and *T. vulpis* identified in dogs showed mitochondrial and SSU rRNA sequence similarity to human isolates, suggesting a potential role of animal reservoirs in the epidemiology of human STHs [26,27].

Recent studies have investigated the prevalence and identification of STH species using molecular techniques, including polymerase chain reaction (PCR) [28–30], real-time PCR [31,32], and loop-mediated isothermal amplification [33,34]. For example, *Ascaris* spp. have been identified using markers such as ITS1/ITS2 regions of rDNA [29,32,35–37], cytochrome c oxidase subunit 1 (*cox1*) [29,38,39], NADH dehydrogenase subunit 1 (*nad1*) [40], and *β-tubulin* [41]. *Trichuris* spp. have been identified and analyzed using 18S rRNA, ITS2 [7], *cox1* [6,42,43], and *β-tubulin* [41]. Mitochondrial genes including *nad1* and *cox1*, known for higher mutation rates, are widely applied to study molecular epidemiology, genetic diversity, and species differentiation. [40,44–49]. In Thailand, a nationwide survey conducted in 2019 reported a helminth infection prevalence of 9.79%, with hookworms, *T*. *trichiura*, *S*. *stercoralis*, and *A*. *lumbricoides* being most common. The southern region showed the highest prevalence (13.43%) [50], suggesting that STH infections remain a significant public health concern in Thailand, particularly in rural and border provinces such as Narathiwat, where close human–animal interactions facilitate ongoing transmission. While several studies have documented the prevalence of STHs in Thailand, molecular epidemiological data remain scarce, particularly in southern provinces where transmission persists. Moreover, few studies in this region have integrated multilocus sequencing approaches that combine ITS1, 18S rRNA gene, and *cox1* markers with population genetic analyses. To address this gap, the present study reports both prevalence estimates and multilocus molecular characterization of STHs in Narathiwat Province, southern Thailand. Specifically, PCR amplification of the ITS1 and 18S rRNA gene regions was employed for species identification and assessment of zoonotic potential, while *cox1* sequencing was used to investigate genetic variation among isolates. By integrating prevalence data with molecular and population-level analyses, this study contributes to the molecular epidemiology of STHs in Thailand and provides deeper insight into parasite diversity, zoonotic potential, and region-specific transmission dynamics.

## Materials and methods

### Ethical approval

This cross-sectional study was conducted using retrospectively analyzed, previously collected fecal samples and associated data from Narathiwat Province, southern Thailand. Archived samples were obtained from the stool bank of the Department of Parasitology, Phramongkutklao College of Medicine, Bangkok, Thailand. The fecal samples and datasets were accessed for research purposes on 1 October 2023. The original collection and storage of fecal samples were approved by the Institutional Review Board of the Royal Thai Army Medical Department (IRBRTA 067/2560) for public health surveillance and research purposes. The protocol for the present study was reviewed and approved by the Institutional Review Board of the Faculty of Medicine, Vajira Hospital, Bangkok, Thailand (COA 150/2566), and was conducted in accordance with internationally recognized ethical principles for research involving human participants, including the Declaration of Helsinki. All samples and datasets used in this study were fully anonymized prior to access by the investigators. The authors did not have access to any information that could directly identify individual participants at any stage of the study. Unique study codes were used in place of personal identifiers, and re-identification of participants was not possible during or after data analysis. As this study involved the secondary use of archived, anonymized samples and data, the requirement for informed consent was waived by the approving ethics committees.

### Fecal sample collection

A total of 210 fecal samples were collected from Narathiwat Province and subsequently stored in the stool bank of the Department of Parasitology, Phramongkutklao College of Medicine. The province is characterized by a tropical climate with high temperatures and humidity throughout the year. These environmental conditions are conducive to the survival and transmission of STHs, supporting completion of their life cycles and sustained transmission within the local population.

### Examination of fecal samples for identifying parasitic infections

All 210 fecal samples were examined for intestinal helminth infections using three parasitological methods: simple smear, Kato–Katz thick smear, and the phosphate-buffered saline (PBS)–ethyl acetate sedimentation technique. The simple smear and Kato–Katz methods were used for direct microscopic detection of helminth eggs, while the PBS–ethyl acetate sedimentation technique served as a concentration method to enhance the recovery of parasite stages. After processing with each of the three techniques, the samples were examined microscopically for the presence of helminth eggs. Parasite prevalence was determined based on the results of these microscopic examinations. Stool samples positive for STHs were subsequently selected for molecular analysis. DNA was extracted from an adequate amount of fecal material obtained from samples positive for *Ascaris* and *Trichuris* spp. following PBS–ethyl acetate sedimentation, which provided a higher concentration of parasite eggs suitable for DNA extraction. Samples negative for STHs or containing other helminth species were not subjected to DNA extraction.

### DNA extraction of the STHs from positive fecal samples

DNA was extracted from each STH-positive sample using 200 µL of the concentrate obtained after PBS–ethyl acetate sedimentation. The samples were homogenized for 1 min and then mixed with 1.4 mL of InhibitEX Buffer. To improve DNA yield, five freeze–thaw cycles were applied using liquid nitrogen  at −196°C to break the egg shells and facilitate genetic material release. DNA was then extracted using the QIAamp Fast DNA Stool Mini Kit (Qiagen GmbH, Hilden, Germany) and eluted in 50 μL of buffer, following the manufacturer’s protocol. The DNA obtained served as a template for PCR, used to identify the STHs.

### PCR targeting the ITS1 region and *cox1* from *Ascaris* spp

The ITS1-based PCR for detecting *Ascaris* spp. was conducted in a total volume of 25 µL. The reaction mix comprised the DNA template, 10 pmol of each primer, and 1X KAPA2G Fast HotStart ReadyMix with dye (Roche, Basel, Switzerland). The primers used were designed by Ishiwata et al. [37]: F2662: 5’-GGCAAAAGTCGTAACAAGGT-3´ as the forward and R3214: 5’-CTGCAATTCGCACTATTTATCG-3´ as the reverse primer. The PCR method was optimized based on the study findings. PCR was conducted using the MasterCycler instrument (Bio-Rad, CA, USA). The amplification program consisted of the following steps; predenaturation at 94°C for 5 min; 35 cycles consisting of denaturation at 94°C for 30 s, annealing at 55°C for 30 s, extension at 72°C for 30 s; a final extension at 72°C for 10 min; and holding at 12°C. The amplicons obtained were ~576 bp long.

PCR for detecting *cox1* in *Ascaris* spp. was performed using the same reagents, machine, and program mentioned above. However, the primers used were As-Co1F: 5′-TTTTTTGGTCATCCTGAGGTTTAT-3′ and As-Co1R: 5′ -ACATAATGAAAATGACTAACA

AC-3′, which were designed by Peng et al. [39]. The amplicons were 431-bp long. Moreover, the PCRs for ITS1 and *cox1* in *Ascaris* spp. were optimized anew in this study.

### PCR for amplifying the 18S rRNA and *cox1* of *Trichuris* spp

PCR amplification of the 18S rRNA gene of *Trichuris* spp. was performed using the same reagents, thermocycler, and cycling conditions described previously. The primers used were designed by Guardone et al. [30], consisting of the forward primer 18S965F (5′-GGCGATCAGATACCGCCCTAGTT-3′) and the reverse primer 18S1573R (5′-TACAAAGGGCAGGGACGTAGT-3′), yielding an amplicon of 724 bp.

Amplification of *cox1* from *Trichuris* spp. was conducted under the same PCR conditions. The primers, developed by Xie et al. [42], comprised a forward primer (5′-AAAAATGGCTATATACAGGT-3′) and a reverse primer (5′-GGGGCCAAATACTTTAAAT-3′), producing an amplicon of 804-bp. PCR cycling parameters for both the 18S rRNA and *cox1* targets of *Trichuris* spp. were re-optimized in this study.

### Sequencing of the *Ascaris* and *Trichuris* spp. PCR products

Amplicons of the *Ascaris* ITS1 region, *Trichuris* 18S rRNA gene, and mitochondrial *cox1* gene from both parasites were excised from 2% agarose gels and transferred to 2 mL microcentrifuge tubes for sequencing. Sequencing was performed at U2Bio (Thailand) Co., Ltd., Bangkok, Thailand, using BTSeq™ sequencing technology. The resulting sequences were aligned using ClustalW implemented in MEGA X software [51]. Species identification and sequence similarity were assessed by comparison with reference sequences deposited in the NCBI database using the Basic Local Alignment Search Tool (BLAST). All generated sequences were submitted to GenBank and subsequently used for phylogenetic analyses of *Ascaris* and *Trichuris* spp.

### Determining the genetic variations in *Ascaris* and *Trichuris* spp

Genetic variation within populations of *Ascaris* and *Trichuris* spp. was assessed using samples that tested positive by PCR for the ITS1, 18S rRNA, and *cox1* markers. Sequence data were analyzed to determine the number of haplotypes, number of segregating sites (polymorphisms), and nucleotide diversity (π) using DnaSP version 5.10.1 [52].

Genetic differentiation based on the ITS1 region of *Ascaris* spp., the 18S rRNA gene of *Trichuris* spp., and the *cox1* gene of both genera was evaluated using pairwise F_ST_ values estimated in DnaSP version 5.10.1 [52]. F_ST_ values ranging from 0.0 to 0.05 indicate minimal genetic variability between populations, suggesting substantial gene flow. Values between 0.05 and 0.15 suggest a moderate level of genetic variation, whereas values greater than 0.15 indicate high levels of genetic differentiation, typically associated with limited or negligible gene flow [53].

### Phylogenetic trees of *Ascaris* and *Trichuris* spp

The nucleotide sequences of the ITS1 and *cox1* genes from *Ascaris* spp. and the 18S rRNA and *cox1* genes from *Trichuris* spp. were aligned using the ClustalW algorithm implemented in MEGA 11 software [51]. Maximum likelihood phylogenetic trees were constructed using the Tamura 3-parameter model in MEGA 11 with 1,000 bootstrap replicates, based on ITS1 sequences of *Ascaris* spp., 18S rRNA sequences of *Trichuris* spp., and *cox1* sequences from both parasites. Reference sequences were retrieved from the GenBank database.

## Results

### Microscopy-based determination of the prevalence of parasitic infections

The microscopic examination of a total of 210 stool samples using the three techniques mentioned previously indicated a parasite prevalence of 14.76% for *Trichuris* spp. (31/210), 6.67% for *Ascaris* spp. (14/210), 4.76% for mixed *Ascaris* and *Trichuris* spp. infections (10/210), 10.00% for *Blastocystis* sp. (21/210), 1.43% for hookworms (3/210), 0.95% for *Entamoeba coli* (2/210), and 0.47% for *Giardia intestinalis* (1/210). Among the fecal samples containing STHs, those with sufficient fecal material for DNA extraction and subsequent PCR analysis were selected for molecular characterization. Of these, 14 samples were positive for *Ascaris* spp., 29 for *Trichuris* spp., and 8 showed mixed infections with both *Ascaris* and *Trichuris* spp. However, three hookworm samples were excluded due to insufficient fecal material for DNA extraction.

### Detection rate of the PCR assays for the ITS1 region and *cox1* for *Ascaris* spp

**Fig 1:**
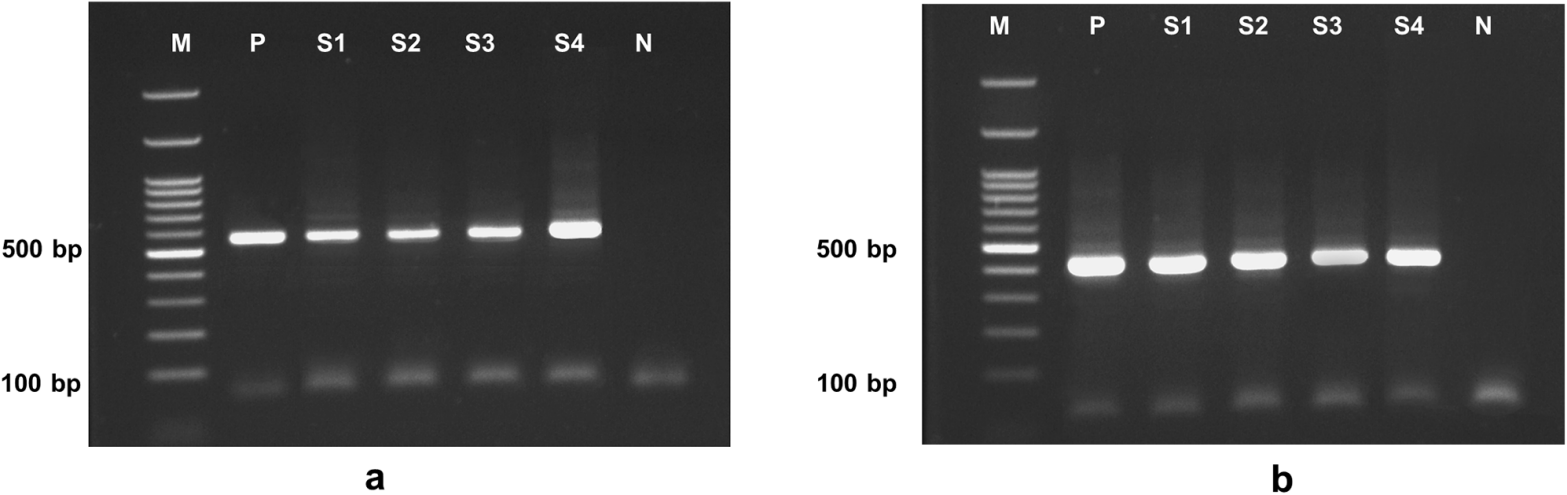
PCR products of the ITS1 (a) and *cox1* (b) genes of *Ascaris* spp. obtained from the Narathiwat Province, southern Thailand. **(1a)** PCR products of *Ascaris* spp. ITS1 were 576-bp long. **(1b)** PCR products of *A. lumbricoides cox1* were 431-bp long. M = 100-bp DNA ladder, P = positive control, N = negative control, and S = fecal sample of an infected person.

The ITS1-PCR assay detected *Ascaris* spp. DNA in 92.86% (13/14) of the fecal samples. The ITS1-PCR amplicons of *A. lumbricoides* and *A. suum* were identical in size, measuring 576- bp (Fig 1a). The ITS1-PCR assay detected *Ascaris* spp. in all eight fecal samples, all of which were mixed infected with *Trichuris* spp., yielding a detection rate of 100% (8/8). The overall detection rate of the ITS1-PCR assay for detecting *Ascaris* spp. was 95.45% (21/22).

The *cox1*-PCR assay used to detect the *cox1* of *Ascaris* spp. demonstrated a detection rate of 85.71% (12/14). The *cox1*-PCR amplicons from *Ascaris* spp. were 431-bp in length. In the eight fecal samples with mixed infections, the *cox1*-PCR assay successfully amplified *cox1* from *Ascaris* spp. with a detection rate of 100% (8/8). The overall detection rate of the *cox1*-PCR assay for *Ascaris* spp. detection was 90.90% (20/22) (Table 1).

**Table 1:** Detection rate of the PCR assay for detecting *Ascaris* spp. and *Trichuris* spp. using fecal samples collected in Narathiwat Province, southern Thailand.

Parasites	Number of samples for PCR assay	PCR detection rate (%)			
		ITS1 (*Ascaris* spp.)	18S rRNA (*Trichuris* spp.)	*cox1* (*Ascaris* spp.)	*cox1* (*Trichuris* spp.)
*Ascaris* spp. only	14	92.86% (13/14)	–	85.71% (12/14)	–
*Trichuris* spp. only	29	–	75.86% (22/29)	–	48.27% (14/29)
Mixed infection of *Ascaris* spp. and *Trichuris* spp.	8	100% (8/8)	75.0% (6/8)	100% (8/8)	50% (4/8)
**Overall presence**					
*Ascaris* spp.	22	95.45% (21/22)	–	90.90% (20/22)	–
*Trichuris* spp.	37	–	75.67% (28/37)	–	48.65% (18/37)

### Detection rate of the PCR assays for detecting 18S rRNA and *cox1* from *Trichuris s*pp

**Fig 2:**
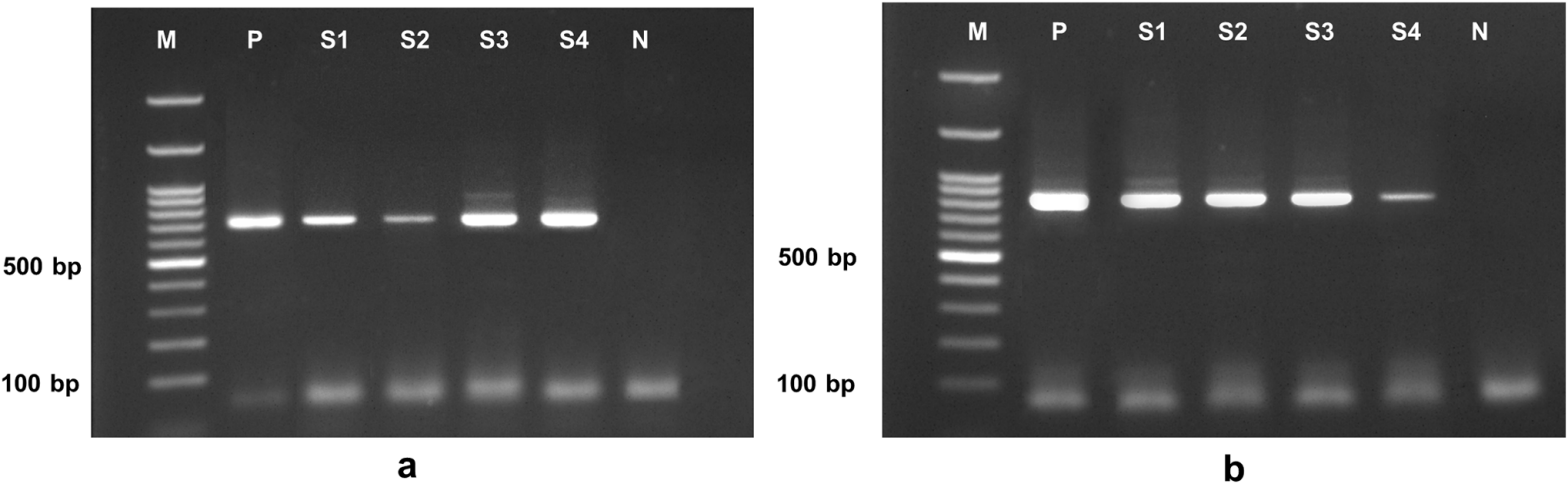
PCR products of the 18S rRNA (a) and *cox1* (b) genes of *Trichuris* spp. obtained from the Narathiwat Province, southern Thailand. **(2a)** PCR products of *Trichuris* spp. 18S rRNA were 724-bp in length. **(2b)** PCR products of *T*. *trichiura cox1* were 804-bp long. M = 100-bp DNA ladder, P = positive control, N = negative control, and S = fecal sample of an infected person.

The PCR assay targeting the 18S rRNA region of *Trichuris* spp. showed a detection rate of 75.86% (22/29), producing amplicons of 724-bp (Fig 2a). Among the eight fecal samples with mixed infections of *Ascaris* spp. and *Trichuris* spp., the 18S rRNA-PCR assay detected *Trichuris* spp. in six samples, corresponding to a detection rate of 75.0% (6/8). The overall prevalence of *Trichuris* spp. was 75.67% (28/37) (Table 2).

The *cox1*-PCR assay targeting *Trichuris* spp. exhibited a detection rate of 48.27% (14/29), with PCR products of 804-bp (Fig 2b). Among the eight fecal samples with mixed infections, the *cox1*-PCR assay detected *Trichuris* spp. in four samples, yielding a detection rate of 50% (4/8). The overall detection rate of the *cox1*-PCR assay for detecting *Trichuris* spp. was 48.65% (18/37) (Table 1).

### Nucleotide sequencing and genetic differentiation of *Ascaris* spp. based on ITS1 and *cox1*

DNA sequencing was performed using parasite eggs recovered from stool samples; therefore, the sequences represent DNA derived from egg populations rather than individual worms. Inspection of the sequencing chromatograms revealed no overlapping peaks, suggesting that each sample was dominated by a single *Ascaris* genotype. From nucleotide sequence analysis, the sequences of 21 ITS1 from *Ascaris* spp. were aligned and designated as isolates Nrtw1–Nrtw21 (Accession numbers: PP758217–PP758237). Among these sequences, two distinctive haplotypes, A and B, were identified. A consisted of 20 isolates, including Nrtw1–Nrtw11 (Accession numbers: PP758217–PP758227), Nrtw13–Nrtw21 (Accession numbers: PP758229– PP758237). Moreover, the nucleotide sequences of A were 99.8%–100% identical to those of *A. lumbricoides*. B contained a single isolate (Nrtw12; accession number PP758228). Additionally, the BLAST analysis of the haplotype B nucleotide sequences demonstrated 100% identity with *A*. *suum* P4CLAOI1 (MF358943) isolated from Laos and Thailand (MF358944). Moreover, among the ITS1 haplotypes (A and B) identified from 21 isolates of *Ascaris* spp., only two polymorphic sites were detected, indicating very low nucleotide diversity (Pi = 0.00035) and low haplotype diversity (Hd = 0.095). In addition, genetic divergence analysis revealed no nucleotide variation within haplotypes, whereas haplotypes A and B were genetically distinct, as evidenced by a pairwise F_ST_ value of 1.000.

In this study, 20 nucleotide sequences for *cox1* were obtained from *Ascaris* spp., designated as isolates NwTh1–NwTh20 (Accession numbers: PP758244–PP758263). They were classified into five haplotypes: D, E, F, G, and H. D comprised three isolates: NwTh1 (PP758244), NwTh5 (PP758248), and NwTh12 (PP758255). E included ten isolates: NwTh2 (PP758245), NwTh11 (PP758254), and NwTh13–NwTh20 (PP758256–PP758263). F contained two isolates: NwTh3 (PP758246) and NwTh4 (PP758247). G consisted of two isolates: NwTh6 (PP758249) and NwTh7 (PP758250). Lastly, H comprised three isolates: NwTh8–NwTh10 (PP758251–PP758253). The haplotypes D, E, and F exhibited 99.77%–100% similarity with *A*. *lumbricoides*, whereas G demonstrated 100% homology with *A*. *suum.* Furthermore, analysis of the *cox1* sequences revealed four polymorphic sites among five haplotypes, resulting in a nucleotide diversity of 0.00404 and a haplotype diversity of 0.72. Pairwise F_ST_ values among the haplotypes were 1.000, indicating complete genetic differentiation among the analyzed haplotypes. Moreover, the *A*. *suum* isolate identified by ITS1-PCR failed to yield any amplification with the *cox1*-PCR assay.

### Nucleotide sequencing and genetic differentiation of *Trichuris* spp. based on 18S rRNA and *cox1*

A total of 28 18S rRNA sequences from *Trichuris* spp. were divided into four haplotypes, designated A, B, C, and D. A was the most prevalent, comprising 18 isolates: TtNw1–TtNw9 (Accession numbers: PP758291–PP758299), TtNw12–TtNw13 (PP758302–PP758303), TtNw22 (PP758312), and TtNw26–TtNw28 (PP758316–PP758318). B included three isolates: TtNw10–TtNw11 (PP758300–PP758301) and TtNw21 (PP758311). C consisted of four isolates: TtNw14–TtNw17 (PP758304–PP758307). D comprised three isolates: TtNw23–TtNw25 (PP758313–PP758315). The nucleotide sequences of A, B, C, and D showed 98.83%–100% similarity with *T*. *trichiura*. Genetic analysis identified four haplotypes with 11 nucleotide polymorphisms, yielding a nucleotide diversity of 0.00309 and a haplotype diversity of 0.563. Analysis of pairwise F_ST_ among haplotypes using the 18S rRNA gene yielded values of 1.0000, consistent with complete genetic isolation among groups.

Based on *cox1* of *Trichuris* spp. a total of 18 sequences were obtained, four haplotypes were identified: E, F, G, and H. E comprised three isolates: NwTh1–NwTh3 (PP758323–PP758325). F included six isolates: NwTh4–NwTh9 (PP758326–PP758331). G consisted of three isolates: NwTh10–NwTh12 (PP758332–PP758334). H contained six isolates: NwTh13–NwTh18 (PP758335–PP758340). The *Trichuris* spp. *cox1* sequences showed 95.27%–98.88% similarity to the *T. trichiura* sequences in the GenBank database. Analysis of the *cox1* sequences revealed four distinct haplotypes characterized by 40 polymorphic nucleotide sites. These variations produced a nucleotide diversity of 0.01639 and indicated substantial haplotype diversity (0.765). Pairwise comparisons among the four haplotypes revealed absolute genetic differentiation (F_ST_ = 1.00000).

### Phylogenetic trees of *Ascaris* spp was constructed based on ITS1 and *cox1*

**Fig 3:**
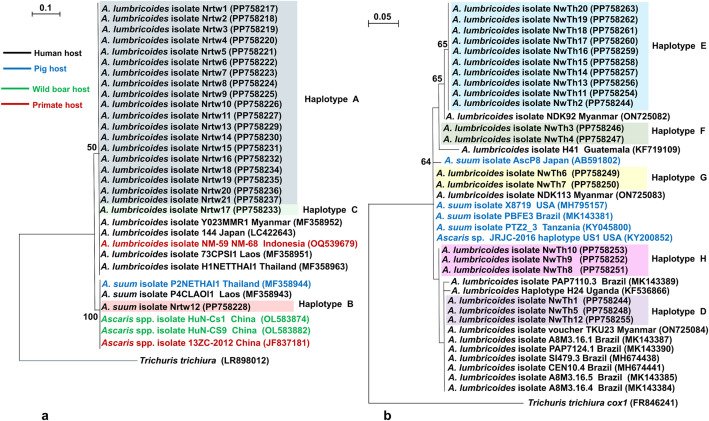
Maximum likelihood phylogenetic trees of *Ascaris* spp. isolates from Narathiwat Province, southern Thailand, based on the ITS1 region and the mitochondrial *cox1* gene. **(3a)** The phylogenetic tree based on the ITS1 region was constructed from a 474-bp alignment without gaps and included 21 isolates from this study (Nrtw1–Nrtw21). Reference sequences of *Ascaris lumbricoides*, *Ascaris suum*, and *Ascaris* spp. retrieved from GenBank were included in the analysis. **(3b)** The phylogenetic tree based on the mitochondrial *cox1* gene was constructed from a 383-bp alignment without gaps and included 20 isolates obtained in this study (NwTh1–NwTh20). Reference sequences of *A. lumbricoides*, *A. suum*, and *Ascaris* spp. from GenBank were also included. For both trees, *Trichuris trichiura* was used as the outgroup. Bootstrap values based on 1,000 replicates are shown at the nodes, with only values >50% displayed. Accession numbers are indicated in parentheses after isolate names. Correspondence between ITS1 and *cox1* isolates is provided in S1 Table.

The phylogenetic tree of *Ascaris* spp., constructed based on the 474-bp ITS1 region, revealed two distinct clades corresponding to *A*. *lumbricoides* and *A*. *suum*. Haplotype A clustered with the *A*. *lumbricoides* isolates from Myanmar (Y023MMR1; humans), Japan (144; humans), Indonesia (NM-59 and NM-68; primate), Laos (73CPSI1; humans), and Thailand (H1NETTHAI1; humans), with bootstrap values of 50%. Notably, haplotype B (isolate Nrtw12) showed a remarkable genetic similarity with the *A*. *suum* isolates P2NETHAI1 (pigs) from Thailand and P4CLAOI1 (humans) from Laos, as well as the *Ascaris* spp. isolates HuN-Cs1 and – HuN-CS9 (wild boars) and 13ZC-2012 (primate), from China, with bootstrap values of 100% (Fig 3a).

The phylogenetic tree of *Ascaris* spp. constructed using the 383-bp *cox1* grouped the isolates into four separate clades. Haplotype D was grouped within the clade containing the *A*. *lumbricoides* isolates from Myanmar (TKU23) and Brazil (A8M3.16.1, PAP7124.1, SI479.3, CEN10.4, A8M3.16.4, and A8M3.16.5). Haplotype E revealed a robust genetic similarity to the *A*. *lumbricoides* isolate NDK92 from Myanmar, as indicated by a bootstrap value of 65%. Haplotype F exhibited a genetic homology with the *A*. *lumbricoides* isolate NDK92 from Myanmar, as suggested by a bootstrap value of 65%. Haplotype G (isolate NwTh6 and NwTh7) shared a close genetic relationship with the *A*. *lumbricoides* isolate NDK113 from Myanmar; the *A*. *suum* isolate AscP8 from Japan, as well as isolates X8719 from the United States, PBFE3 from Brazil, PTZ2_3 from Tanzania and the *Ascaris* spp. isolate JRJC-2016 haplotype US1 from the United States. This result suggests that haplotype G may have originated through hybridization or shared ancestry. In addition, Haplotype G showed different phylogenetic placements depending on the genetic marker analyzed. Based on the nuclear ITS1 sequences, haplotype G clustered within the same clade as reference sequences of *A. lumbricoides*. In contrast, phylogenetic analysis of *cox1* gene placed these isolates within the *A. suum* clade. This discordant clustering between ITS1 and *cox1* markers may indicate genetic incongruence between the two loci. In addition, haplotype H showed a close phylogenetic relationship with haplotype D and other isolates obtained from humans belonging to various geographical regions, including Brazil (PAP7110.3, A8M3.16.1, PAP7124.1, SI479.3, CEN10.4, A8M3.16.5, and A8M3.16.4), Myanmar (TKU23), and Uganda (H24) (Fig 3b). Moreover, amplification of *cox1* gene was unsuccessful for isolate Nrtw12; therefore, no mitochondrial sequence was obtained for comparison with the ITS1 results (S1 Table).

### Phylogenetic trees of *Trichuris* spp. was constructed based on 18S rRNA and *cox1*

**Fig 4:**
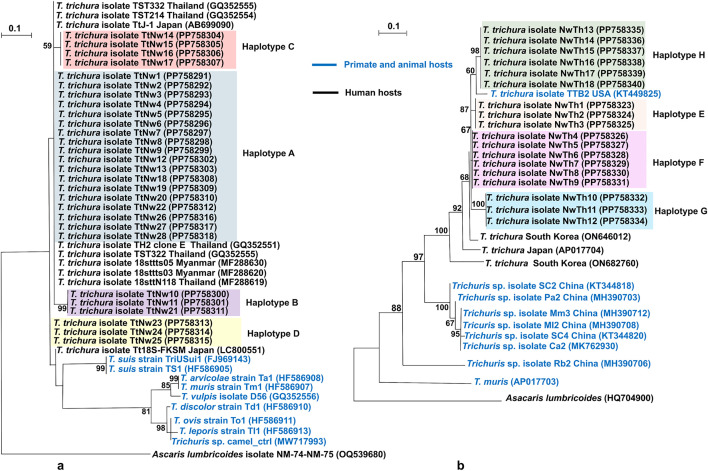
Maximum likelihood phylogenetic trees of *Trichuris* spp. isolated from Narathiwat Province, southern Thailand, inferred from 18S rRNA and *cox1* gene sequences. **(4a)** Phylogenetic tree based on the 18S rRNA gene constructed from a 691-bp alignment without gaps, including 28 isolates from Narathiwat Province, Thailand (TtNw1–TtNw28). Reference sequences of *Trichuris* spp. retrieved from GenBank were used for comparison. **(4b)** Phylogenetic tree based on the mitochondrial *cox1* gene constructed from a 767-bp alignment without gaps, including 18 isolates from Narathiwat Province (NwTh1–NwTh18). Reference sequences of *T. trichiura* and other *Trichuris* spp. from GenBank were also analyzed. In both trees, isolates derived from animal hosts are highlighted in blue, and *Ascaris lumbricoides* was used as the outgroup. Bootstrap values based on 1,000 replicates are shown at the nodes, with only values >50% displayed. Accession numbers are indicated in parentheses after isolate names. Correspondence between isolates analyzed for the 18S rRNA and *cox1* genes is provided in S2 Table.

A phylogenetic tree based on the 691-bp fragment of 18S rRNA gene of *T. trichiura* revealed four groups. Haplotype A clustered with the *T*. *trichiura* isolate TtJ-1 from Japan; TST214, TST332, and TH2 clone E from Thailand; and 8sttN118, 18sttts03, and 18sttts05 from Myanmar, as well as the reference sequence (GenBank accession no. DQ118536). In contrast, haplotype B appeared to have diverged from haplotype A, with strong bootstrap support (99%). Additionally, haplotype C showed a separation from haplotype A with moderate bootstrap support (59%). Moreover, haplotype D showed robust genetic similarity to the *T*. *trichiura* isolate Tt18S-FKSM from Japan (Fig 4a).

A phylogenetic tree constructed using the 767-bp *cox1* sequence divided *T. trichiura* into four clades. Haplotype E clustered as a distinct lineage with 87% bootstrap support, while haplotype F clustered closely with a *T. trichiura* isolate from South Korea (ON646012). Interestingly, haplotype G was unique from the others, with a robust bootstrap support of 100%. Similarly, haplotype H was separated from the remaining haplotypes with 98% bootstrap support and clustered closely with the *T. trichiura* isolate TTB2 from the United States (Fig 4b).

## Discussion

STH infections are highly prevalent in southern Thailand, particularly in the Narathiwat Province. The tropical rainforest climate, with consistently high humidity, warm temperatures, and abundant year-round rainfall, creates conditions ideal for the survival and development of STH eggs and larvae in the soil. However, molecular epidemiological data on these parasites in this region remain limited. Such data are crucial for a comprehensive understanding of helminth population dynamics and potential zoonotic transmission pathways. Molecular analysis of the ITS1 region revealed that most fecal samples contained *A. lumbricoides*, showing 100% sequence identity with isolates NM-59 and NM-68 previously reported from Sumatran orangutans (*Pongo abelii*) in Indonesia [54]. This finding suggests that *A*. *lumbricoides* may have a broad host range, including humans and primates. In addition, haplotype B was identified as *A*. *suum*, differing by four polymorphic sites, a finding which was consistent with those of a previous study reporting minimal genetic divergence (1.3%) within the ITS1 region of *A*. *lumbricoides* isolates from humans and *A*. *suum* from pigs [55]. The *A*. *suum* isolate was genetically 100% identical with P4CLAOI1 from Laos and P2NETHAI1 from northeastern Thailand, suggesting this haplotype to be widely distributed across Thailand. Unfortunately, the fecal sample positive for *A*. *suum* detected utilizing the ITS1-PCR assay could not be used for amplifying *cox1*, owing to which a mitochondrial profile for comparison with other haplotypes was not obtained. Nonetheless, ITS1-based phylogenetic analysis of *Ascaris* spp. revealed two distinct clades corresponding to *A*. *lumbricoides* and *A*. *suum*, consistent with previous findings [44,45,56]. *A*. *lumbricoides* haplotype A clustered with isolates derived from humans and nonhuman primate hosts, indicating the potential for zoonotic transmission. Similarly, the *A*. *suum* isolate Nrtw12 clustered with *Ascaris* derived from humans, pigs, wild boars, and primates in China, implying possible cross-species transmission. As the samples were collected from Islamic communities, alternative non-pig hosts should be considered. Phylogenetic analysis using *cox1* revealed the *A*. *lumbricoides* haplotypes D, E, F, and G to be closely related to global human isolates, indicating the capacity for widespread transmission and gene flow. Notably, haplotype G (isolate NwTh6, and NwTh7) clustered with both human-derived *A. lumbricoides* and pig-derived *A. suum*, suggesting a possible hybrid origin [28,29,43–45,57]. These findings underscore the complex genetic structure of *Ascaris* populations. Although the mitochondrial genomes of *A*. *lumbricoides* and *A*. *suum* vary by only 1.9%, recent genomic analyses have revealed marked genetic variations between the two species. Furthermore, *A*. *lumbricoides* may represent a more ancestral lineage within the genus [44,45,49]. In the present study, discordant species identification between ITS1 and *cox1* genes was observed in some samples in this study. Similar inconsistencies have been reported previously in molecular studies of *Ascaris* populations and may reflect genetic exchange between *A. lumbricoides* and *A. suum*. Because mitochondrial DNA is maternally inherited whereas nuclear markers are inherited biparentally, hybridization or introgression between these taxa may produce discordant phylogenetic signals. Alternatively, this pattern may also result from incomplete lineage sorting or mixed infections in which one genotype predominates in the sequencing chromatograms. The close evolutionary relationship between these taxa has also resulted in overlapping mitochondrial haplotypes, making species discrimination based solely on mitochondrial markers challenging. Therefore, multilocus approaches are recommended to further clarify the genetic relationships within the *Ascaris* species complex. This situation may be particularly relevant in Thailand, where close contact between humans and pigs in rural communities may facilitate cross-transmission of *Ascaris* species, potentially contributing to ongoing gene flow and introgression between parasite populations infecting different hosts. Although morphological, genetic, and experimental infection data indicate that these parasites may represent host-adapted variants, their taxonomic status as a single species or distinct species remains debated. In addition, pairwise F_ST_ analysis revealed substantial genetic differentiation among *A. lumbricoides* populations in Narathiwat Province, which may be influenced by environmental conditions, host-related factors, or gene flow among parasite populations.

In *Trichuris* spp., the 18S rRNA gene is highly conserved and thus is widely used for phylogenetic studies and to distinguish higher taxonomic levels [30,58]. The distinct separation between *T. trichiura* from the Narathiwat Province and animal-derived *Trichuris* spp. indicates a clear genetic divergence between human and nonhuman hosts. Haplotype A (18 isolates) was predominant and closely related to isolates from Thailand, Myanmar, and Japan, suggesting it to be a predominant or widespread lineage. In contrast, haplotype B formed a well-supported distinct clade (99% bootstrap), implying that it forms a genetically distinct group possibly shaped by geographic, ecological, or host-specific factors. These findings support the utility of the 18S rRNA gene for species-level differentiation within *Trichuris* spp. [30,58]. The cox1 sequences of *T. trichiura* from the Narathiwat Province revealed notable genetic diversity, forming four distinct haplotypes (E, F, G, and H) with strong clade support (Fig 4b). Haplotypes E, F, and G were closely related to the T. trichiura isolates, collected from South Korea, Japan, and the USA populations, suggesting shared lineage. Notably, haplotype H exhibited genetic similarity to *Trichuris* spp. from primates (Papio anubis) in the USA, indicating a possible zoonotic link and cross-species transmission potential. The mitochondrial *cox1* gene showed adequate sequence polymorphism and is therefore an appropriate molecular marker for the analysis of genetic diversity in *Trichuris* spp. A combined analysis of 18S rRNA and cox1 revealed marked genetic variations and complex phylogenetic relationships, supported by pairwise statistics and tree topology. These findings indicate that the *T*. *trichiura* population in Narathiwat exhibits evolutionary divergence that may affect transmission patterns, host specificity, and parasite adaptation, which are important for epidemiology and control strategies.

Our findings reveal that human-derived *Ascaris* haplotypes in Narathiwat are genetically similar to pig-associated isolates, consistent with reports from Thailand and China, where human and pig isolates often cluster within shared mitochondrial clades, indicating potential cross-infection or retention of ancestral lineages [25,59,60]. In contrast, *T. trichiura* exhibits distinct human-specific lineages that cluster across geographically diverse human populations and certain non-human primates, remaining separate from pig-derived *T. suis* lineages [7,42,61]. These results underscore the importance of zoonotic reservoirs and lineage specificity in STH transmission and highlight the contribution of Southeast Asian data to the global understanding of STH dynamics. In addition, human trichuriasis in Thailand has historically been attributed solely to *T. trichiura*, and diagnosis in most epidemiological surveys relies primarily on the microscopic identification of characteristic eggs in fecal samples. However, due to the morphological similarity between *T. trichiura* and *T. incognita*, the presence of cryptic or genetically distinct *Trichuris* species cannot be completely excluded. To date, there has been no confirmed report of *T. incognita* in Thailand. Nevertheless, the increasing application of molecular tools, such as PCR amplification and sequencing of ribosomal and mitochondrial markers (e.g., ITS regions and *cox1*), has revealed substantial genetic diversity within *Trichuris* populations infecting humans and non-human primates in different regions of the world. Therefore, further molecular investigations of *Trichuris* isolates from Thailand are warranted to determine whether cryptic species occur and to evaluate their potential implications for diagnosis, epidemiology, and the effectiveness of anthelmintic treatment. Moreover, to fully capture the genetic diversity and population structure of STHs in southern Thailand, additional investigations in other endemic areas are needed. Evaluating STH prevalence in domestic animals is essential to clarify zoonotic transmission pathways. Molecular approaches provide precise species identification, detect mixed infections, and reveal spatial genetic variation, offering critical insights to inform targeted and effective control strategies.

## Conclusion

*Ascaris* and *Trichuris* spp. are the predominant STHs in Narathiwat, southern Thailand. This study provides novel molecular information on these parasites from this region. Molecular analysis revealed the presence of *A*. *lumbricoides* and *A*. *suum*, with the former showing genetic similarity to human and primate isolates, and the latter clustered with isolates from humans, pigs, primates, and wild boars. Analysis of the *A*. *lumbricoides cox1* indicated genetic variations and potential overlap with *A*. *suum*. The molecular characterization of *Trichuris* species identified only *T*. *trichiura* in the samples. Analysis of the 18S rRNA and *cox1* sequences of *T*. *trichiura* revealed evident genetic variations among the isolates. Interestingly, the *cox1* sequences from human-derived *T*. *trichiura* were genetically close to isolates from olive baboons, suggesting host-sharing during evolution or cross-species transmission between humans and nonhuman primates. Significant genetic differentiation among populations in both species may reflect genetic structuring influenced by geographic or ecological factors. These findings enhance our understanding of the transmission dynamics and genetic diversity of STHs in southern Thailand, informing targeted control strategies. In addition, sustainable reduction of soil-transmitted helminthiasis in Narathiwat requires implementation of integrated strategies combining culturally tailored education, improved sanitation, molecular surveillance, and community engagement.

## Supporting information

S1 Table Correspondence between ITS1 and *cox1* isolates of *Ascaris* spp. analyzed in this study. (PDF)

S2 Table Correspondence between 18S rRNA and *cox1* isolates of *Trichuris* spp. analyzed in this study. (PDF)
