# Supplementary material for: Molecular characterization of the prevalent soil-transmitted helminths in Narathiwat Province, southern Thailand
Source: PLoS One. 2026 Apr 16;21(4):e0347339. doi: 10.1371/journal.pone.0347339 (PMC13086308; doi:10.1371/journal.pone.0347339)
Supplement: S1 Table — (PDF) [file pone.0347339.s001.pdf]

Table S1. Correspondence between ITS1 and *cox1* isolates of *Ascaris* spp. analyzed in this study.

Sample ID	ITS1 isolate	*cox1* isolate	Remark
Sample 1	Nrtw1	NwTh1	–
Sample 2	Nrtw2	NwTh2	–
Sample 3	Nrtw3	NwTh3	–
Sample 4	Nrtw4	NwTh4	–
Sample 5	Nrtw5	NwTh5	–
Sample 6	Nrtw6	NwTh6	–
Sample 7	Nrtw-7	NwTh7	–
Sample 8	Nrtw8	NwTh8	–
Sample 9	Nrtw-9	NwTh9	–
Sample 10	Nrtw-10	NwTh10	–
Sample 11	Nrtw11	NwTh11	–
Sample 12	Nrtw12	–	*cox1* amplification failed
Sample 13	Nrtw13	NwTh12	–
Sample 14	Nrtw14	NwTh13	–
Sample 15	Nrtw15	NwTh14	–
Sample 16	Nrtw16	NwTh15	–
Sample 17	Nrtw17	NwTh16	–
Sample 18	Nrtw18	NwTh17	–
Sample 19	Nrtw19	NwTh18	–
Sample 20	Nrtw20	NwTh19	–
Sample 21	Nrtw21	NwTh20	–
