# Supplementary material for: Molecular characterization of the prevalent soil-transmitted helminths in Narathiwat Province, southern Thailand
Source: PLoS One. 2026 Apr 16;21(4):e0347339. doi: 10.1371/journal.pone.0347339 (PMC13086308; doi:10.1371/journal.pone.0347339)
Supplement: S2 Table — (PDF) [file pone.0347339.s002.pdf]

Table S2. Correspondence between 18S rRNA and *cox1* isolates of *Trichuris* spp. analyzed in this study.

Sample ID	18S rRNA isolate	*cox1* isolate	Remark
Sample 1	TtNw1	NwTh1	–
Sample 2	TtNw2	NwTh2	–
Sample 3	TtNw3	NwTh3	–
Sample 4	TtNw4	–	*cox1* amplification failed
Sample 5	TtNw5	–	*cox1* amplification failed
Sample 6	TtNw6	NwTh4	–
Sample 7	Nrtw7	NwTh5	–
Sample 8	TtNw-8	NwTh6	–
Sample 9	TtNw9	-	*cox1* amplification failed
Sample 10	TtNw10	NwTh7	–
Sample 11	TtNw-11	NwTh8	–
Sample 12	TtNw12	NwTh9	*cox1* amplification failed
Sample 13	TtNw13	–	*cox1* amplification failed
Sample 14	TtNw14	NwTh10	–
Sample 15	TtNw15	NwTh11	–
Sample 16	TtNw16	NwTh12	–
Sample 17	TtNw17	–	*cox1* amplification failed
Sample 18	TtNw18	–	*cox1* amplification failed
Sample 19	TtNw19	NwTh13	–
Sample 20	TtNw20	NwTh14	–
Sample 21	TtNw21	–	*cox1* amplification failed
Sample 22	TtNw22	NwTh15	–
Sample 23	TtNw23	–	*cox1* amplification failed
Sample 24	TtNw24	–	*cox1* amplification failed
Sample 25	TtNw25	–	*cox1* amplification failed
Sample 26	TtNw26	NwTh16	–

**Sample ID**	**ITS1 isolate**	***cox1* isolate**	**Remark**
Sample 27	TtNw27	NwTh17	–
Sample 28	TtNw28	NwTh18	–
